# New York Homœopathic Union

**Published:** 1891-07

**Authors:** L. M. Stanton

**Affiliations:** Secretary; 71 West Eighty-eighth Street, New York


### THE NEW YORK HOMŒOPATHIC UNION.

### MINUTES OF MEETING OF APRIL 23D, 1891.

A meeting of the Union was held March 19th at the office of Dr. Carleton, 53 West Forty-fifth Street.

Sections 82–94 of *The Organon* were read. Attention was called to an error in Section 86 of Stratton's translation. It occurs in the sentence "and in what *part* of the body was it (the pain) the worst," which should read, "and in what *position* of the body was it the worst." It was thought that these sections which deal with the taking of the case were more frequently transgressed than any others of *The Organon.*

Hahnemann advises, in Section 91, in those cases where the true picture of disease is masked by drug symptoms, to wait a few days before giving medicine. This seems in many cases the only thing to do, but often the totality of symptoms may at once be prescribed for, thus antidoting those symptoms due to previous medication, and at the same time clearing up the case.

As regards the aggravation of high potencies, which subject was next discussed, it was thought that the aggravation might sometimes be avoided by giving a very minute dose, for instance one No. 10 pellet. Other members thought that with the high potencies the size of the dose made little difference, but that too frequent repetition was the chief factor in the aggravation.

The aggravation of a high potency might be antidoted by a higher potency of the same remedy. Lycopodium was cited as the drug that is more apt to aggravate, in a high potency, than any other.

Dr. Young gave his experience of an extreme sensitiveness to drugs, when in ill health some years ago. At that time, he said, he could get a proving of any drug by holding a vial containing the drug in his hand for a short time, and such a proving that would enable him to say positively what medicine he held. He could distinguish one metal from another by simply holding it for a few minutes between his fingers. By feeling a patient's pulse he could obtain a fair picture of the patient's condition.

This hyper-sensitiveness of the nervous system, which at first was an amusing novelty and promised to be of much value, proved shortly most annoying to Dr. Young. The condition was to a great extent corrected by Agaricus-muscarius.

### Minutes of Meeting of May 21st, 1891.

The previous meeting of the Union was held April 23d, at Dr. Carleton's office, 53 West Forty-fifth Street. In the absence of Dr. Fincke, Dr. Morgan occupied the chair.

The minutes of the previous meeting were read, and Dr. Young's peculiar sensitiveness to drugs was further commented upon. Dr. Morgan said he remembered a series of experiments some years ago which showed how very susceptible some persons are to certain drugs. Potentized Ipecac. in a vial hermetically sealed caused a severe attack of asthma in a person experimented upon, and musk used in the same way produced frequent attacks of fainting in a woman.

Sections 95–103 of *The Organon* were read.

Is there any medicine of value in the prophylaxis of measles? was asked.

Pulsatilla was suggested as useful in many cases, and Swan's Morbilin $^{mmm}$, given three times daily, was thought to be of service in shortening the disease or making it lighter.

Hahnemann, speaking of the epidemic diseases in Section 100, says that each epidemic must be separately and carefully explored, "as every prevailing pestilence is in many regards a phenomenon of its own kind, and on exact examination will be found to be very different from all other former pestilences." But he adds, "excepting epidemics from the same identical tinder of contagion, the small-pox, measles, etc." This section, which gives the idea that we need not individualize in these cases, was objected to, on the ground that this advice leads to routine and ruts; we need to individualize in small-pox, measles, etc., as in any other disease.

Dr. Carleton said that Hahnemann could not have intended to encourage carelessness. In Hahnemann's day most cases in any one epidemic were pretty much all alike. The cases did not differ as at the present time; then these diseases approached the universal type, and on this account Hahnemann was able to suggest those three remedies for cholera which proved so successful in its treatment.

Dr. Morgan said that many years ago "Nux and Salt," one of Humphrey's first specifics, were prescribed for many cases of intermittents, and with success at that time, but to-day we cannot make the exceptions Hahnemann speaks of in Section 100.

Section 102 suggests the importance of writing down the symptoms of our cases, and this cannot be too strongly insisted upon. Dr. Bayard was cited as one who did not take notes, but *he* had an exceptional memory and a profound knowledge of materia medica, and although such a benefit to humanity, his experience has been of little use to the profession.

Incidentally a written record of a case is often useful in proving to a patient how much better he is than he is willing to acknowledge, as he may belong to that class which is never any better until entirely well.

Dr. Morgan spoke very decidedly upon that subject of which we can never hear too much—the materia medica; we cannot study it too carefully. Diagnosis, although important, must always be subordinated to materia medica.

About thirty years ago there was a disease prevalent through Central New York known as the Albany throat disease, having originated in that city.

By the symptoms, subjective and objective, Dr. Morgan was able to select the simillimum and cured his cases while the old-school doctors were speculating upon its etiology or making a diagnosis.

This Albany throat disease is now known as diphtheria.

L. M. STANTON, *Secretary*.

71 West Eighty-eighth Street, New York.

